# *Pglyrp*-Regulated Gut Microflora *Prevotella falsenii*, *Parabacteroides distasonis* and *Bacteroides eggerthii* Enhance and *Alistipes finegoldii* Attenuates Colitis in Mice

**DOI:** 10.1371/journal.pone.0146162

**Published:** 2016-01-04

**Authors:** Roman Dziarski, Shin Yong Park, Des Raj Kashyap, Scot E. Dowd, Dipika Gupta

**Affiliations:** 1 Indiana University School of Medicine–Northwest, Gary, Indiana, United States of America; 2 MR DNA Molecular Research LP, Shallowater, Texas, United States of America; Massachusetts General Hospital, UNITED STATES

## Abstract

Dysbiosis is a hallmark of inflammatory bowel disease (IBD), but it is unclear which specific intestinal bacteria predispose to and which protect from IBD and how they are regulated. Peptidoglycan recognition proteins (*Pglyrps*) are antibacterial, participate in maintaining intestinal microflora, and modulate inflammatory responses. Mice deficient in any one of the four *Pglyrp* genes are more sensitive to dextran sulfate sodium (DSS)-induced colitis, and stools from *Pglyrp*-deficient mice transferred to wild type (WT) germ-free mice predispose them to much more severe colitis than stools from WT mice. However, the identities of these *Pglyrp*-regulated bacteria that predispose *Pglyrp*-deficient mice to colitis or protect WT mice from colitis are not known. Here we identified significant changes in β-diversity of stool bacteria in *Pglyrp*-deficient mice compared with WT mice. The most consistent changes in microbiome in all *Pglyrp*-deficient mice were in *Bacteroidales*, from which we selected four species, two with increased abundance (*Prevotella falsenii* and *Parabacteroides distasonis*) and two with decreased abundance (*Bacteroides eggerthii* and *Alistipes finegoldii*). We then gavaged WT mice with stock type strains of these species to test the hypothesis that they predispose to or protect from DSS-induced colitis. *P*. *falsenii*, *P*. *distasonis*, and *B*. *eggerthii* all enhanced DSS-induced colitis in both WT mice with otherwise undisturbed intestinal microflora and in WT mice with antibiotic-depleted intestinal microflora. By contrast, *A*. *finegoldii* (which is the most abundant species in WT mice) attenuated DSS-induced colitis both in WT mice with otherwise undisturbed intestinal microflora and in WT mice with antibiotic-depleted intestinal microflora, similar to the colitis protective effect of the entire normal microflora. These results identify *P*. *falsenii*, *P*. *distasonis*, and *B*. *eggerthii* as colitis-promoting species and *A*. *finegoldii* as colitis-protective species.

## Introduction

Innate immunity not only defends the host against infections, controls inflammation, and initiates adaptive immunity, but also regulates and maintains normal microflora (microbiome) [1]. This microflora contributes to proper development of the host and determines the host’s sensitivity to many diseases, including inflammatory bowel disease (IBD), allergies, cancer, and obesity. One family of innate immunity proteins that regulate host’s microbiota in both invertebrates and vertebrates is peptidoglycan recognition proteins (PGRPs or *Pglyrps*) [1].

Pglyrps are innate immunity proteins that are conserved from insects to mammals, recognize bacterial cell wall component peptidoglycan, and are antibacterial [1–3]. Mammals have four *Pglyrp* genes, *Pglyrp1*, *Pglyrp2*, *Pglyrp3*, and *Pglyrp4* [4, 5]. *Pglyrp1* is mainly expressed in polymorphonuclear leukocytes, whereas *Pglyrp2*, *Pglyrp3*, and *Pglyrp4* are expressed in epithelial cells of many organs, including salivary glands, throat, tongue, esophagus, stomach, intestine, and skin [5–7]. All mammalian Pglyrps are secreted proteins and are bactericidal for both Gram-positive and Gram-negative bacteria [6, 8–11]. Pglyrp2 is also an amidase that hydrolyzes bacterial cell wall peptidoglycan [12]. *Pglyrps* participate in maintaining normal bacterial flora in the gut [13, 14] and in modulating inflammatory responses to bacteria, allergens, and injury [13–20].

Mice deficient in any one of the four *Pglyrp* genes are more sensitive to dextran sulfate sodium (DSS)-induced colitis, which implies that the presence of *Pglyrps* protects wild type (WT) host from colitis [13]. *Pglyrp1*^-/-^, *Pglyrp2*^-/-^, *Pglyrp3*^-/-^, and *Pglyrp4*^-/-^ mice all have significant and distinct changes in the abundance of several of the eight major groups of *Eubacteria* in their stools, and their increased sensitivity to severe DSS-induced colitis can be transferred to WT germ-free mice by the intestinal microflora from *Pglyrp*-deficient mice [13, 14]. These results suggest that the lack of any one of the four *Pglyrps* results in the development of colitis-predisposing intestinal microflora. However, the exact changes in the intestinal microflora at all taxonomic levels in *Pglyrp*-deficient mice are not known, and also the identities of these *Pglyrp*-regulated bacteria that predispose *Pglyrp*-deficient mice to colitis or protect WT mice from colitis are not known.

Here we first identified which colonic bacteria are changed in *Pglyrp*-deficient mice compared with WT mice. Then, we tested the hypothesis that specific intestinal bacteria that are more abundant in *Pglyrp*-deficient mice enhance DSS-induced colitis, and that intestinal bacteria that are more abundant in WT mice attenuate colitis. We identified three *Bacteroidales* species that enhance DSS-induced colitis in WT mice and one *Bacteroidales* species that attenuates DSS-induced colitis in mice, similar to the protection by normal intestinal microflora. These results identify novel colitis-promoting and colitis-protecting bacteria and show that normal undisturbed intestinal microflora protects from colitis.

## Results

### *Pglyrp*-deficient mice have significant changes in β-diversity of their stool microbiome

Although we previously demonstrated several significant changes in the abundance of major bacterial groups in the stools of *Pglyrp*-deficient mice compared with WT mice [13, 14], the *Pglyrp*-controlled changes in gut microbiota at all taxonomic levels are not known. To determine the diversity of intestinal bacteria in *Pglyrp*-deficient mice and to identify in detail how their intestinal microflora differs from WT mice, we isolated DNA from stool microflora and performed genetic phylotyping (community profiling) using pyrosequencing of the variable regions of bacterial 16S ribosomal RNA (rRNA) genes.

We first evaluated α-diversity of the microbiomes. We identified a total of 378 species and 7545 operational taxonomic units (OTUs) in the stools of all strains: 239, 255, 203, 228, and 202 species and 5196, 5501, 4757, 4864, and 4916 OTUs in WT, *Pglyrp1*^-/-^, *Pglyrp2*^-/-^, *Pglyrp3*^-/-^, and *Pglyrp4*^-/-^ mice, respectively. The total number of OTUs identified in *Pglyrp1*^-/-^ mice was significantly higher than in WT mice, whereas the total numbers of species in *Pglyrp2*^-/-^ and *Pglyrp4*^-/-^ mice and the total numbers of OTUs in *Pglyrp2*^-/-^, *Pglyrp3*^-/-^, and *Pglyrp4*^-/-^ mice were significantly lower than in WT mice (S1 Fig). The numbers of species and OTUs per mouse (microbiome richness) were similar for WT and *Pglyrp*-deficient mice, except for *Pglyrp1*^-/-^ mice, which were significantly higher. The Shannon diversity indices (H) for the species were significantly lower in *Pglyrp1*^-/-^ and *Pglyrp4*^-/-^ than in WT mice, and the Shannon equitability index (E_H_) for the species was lower in *Pglyrp1*^-/-^ than in WT mice and similar for other *Pglyrp*-deficient strains (S1A Fig). The Shannon diversity indices (H) and equitability indices (E_H_) for OTUs were similar for WT and *Pglyrp*-deficient strains (H = 6.34 ± 0.07, 6.44 ± 0.12, 6.42 ± 0.06, 6.39 ± 0.05, 6.34 ± 0.04, and E_H_ = 0.87 ± 0.01, 0.87 ± 0.01, 0.88 ± 0.01, 0.87 ± 0.01, 0.87 ± 0.01).

**Fig 1:**
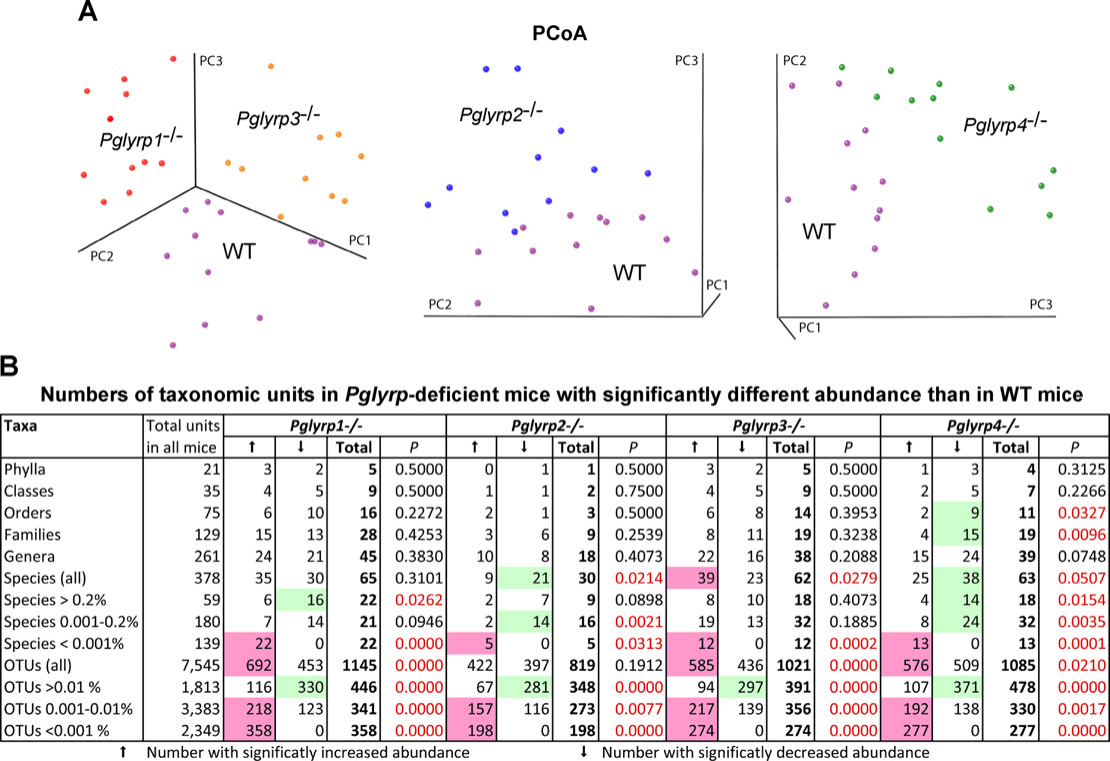
β-diversity in stool microflora in WT and *Pglyrp*-deficient mice. (**A**) Principal Coordinate Analysis (PCoA) by UniFrac (unweighted) of microbiomes in WT and *Pglyrp*-deficient mice. Separation of stool microbiomes of WT mice from stool microbiomes of *Pglyrp1*^-/-^ and *Pglyrp3*^-/-^ mice (left panel), *Pglyrp2*^-/-^ mice (middle panel), and *Pglyrp4*^-/-^ mice (right panel). Each dot corresponds to a stool microbiome from one mouse. (**B**) Numbers of taxonomic units in *Pglyrp*-deficient mice with significantly (at *P*<0.05) increased or decreased abundance compared with WT mice; *P*, significance of the differences between the numbers of significantly increased and significantly decreased taxonomic units; groups with significantly more decreased than increased taxa are shaded in green, and groups with significantly more increased than decreased taxa are shaded in purple. *N* = 12 mice/strain. The % abundance of significantly changed microflora, *P* for abundance in *Pglyrp*-deficient *versus* WT microflora, and the ratio of abundance in *Pglyrp*-deficient to WT microflora for each strain and taxonomic unit are shown in S1–S6 Tables.

We then evaluated β-diversity of the microbiomes. Microfloras from stools of each *Pglyrp*-deficient strain separated from WT microflora in the Principal Coordinate Analysis (PCoA), with microfloras from *Pglyrp1*^-/-^ and *Pglyrp3*^-/-^ mice showing the most distinct separation from the microflora in WT mice (Fig 1A). These results suggested differences in β-diversity in intestinal microbiomes between WT and *Pglyrp*-deficient mice and that microbiomes in each of the *Pglyrp*-deficient strains of mice are distinct and different from WT mice. These results prompted us to further identify the differences between these microbiomes.

We detected many statistically significant differences in bacterial β-diversity between WT and *Pglyrp*-deficient mice. *Pglyrp1*^-/-^, *Pglyrp2*^-/-^, *Pglyrp3*^-/-^, and *Pglyrp4*^-/-^ mice all had significant changes in the abundance of various bacterial groups in the stools at all taxonomic levels (Fig 1B and S1–S6 Tables). While the abundance of some taxonomic groups decreased and others increased, in general there was a statistically significant decrease in the more abundant species and OTUs in *Pglyrp*-deficient mice compared with WT mice, and statistically significant increase in the very low abundant species and OTUs (Fig 1B).

**Fig 2:**
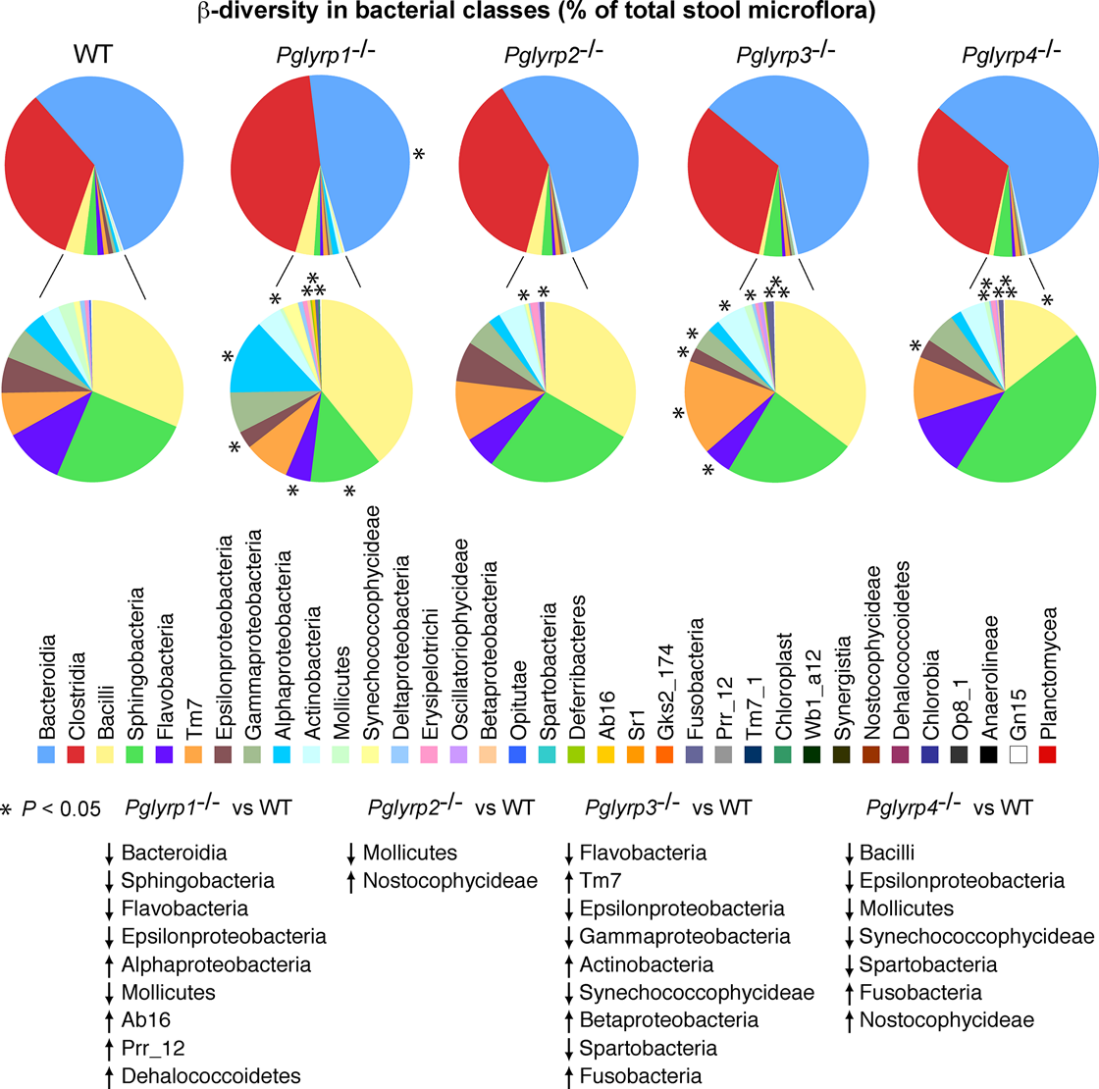
β-diversity in bacterial classes in stool microflora of WT and *Pglyrp*-deficient mice. Class abundance is expressed as % of total stool microflora. The classes with significantly different abundance in *Pglyrp*-deficient *versus* WT mice are marked with an asterisk (*) and listed as significantly (at *P*<0.05) increased or decreased (marked by upward or downward arrows); *N* = 12 mice/strain. The % abundance of significantly changed microflora, *P* for abundance in *Pglyrp*-deficient *versus* WT microflora, and the ratio of abundance in *Pglyrp*-deficient to WT microflora for each strain of mice are shown in S1 Table.

At all taxonomic levels, some changes were common to all or some *Pglyrp*-deficient mice, whereas others were unique for each *Pglyrp*-deficient strain (Fig 1B and S1–S6 Tables). For example, at the class level, the abundance of *Fusobacteria* was significantly increased in *Pglyrp3*^-/-^ and *Pglyrp4*^-/-^ mice (Fig 2 and S1 Table), whereas there was a significantly reduced abundance of *Epsilonproteobacteria* in *Pglyrp1*^-/-^, *Pglyrp3*^-/-^, and *Pglyrp4*^-/-^ mice, of *Mollicutes* in *Pglyrp1*^-/-^, *Pglyrp2*^-/-^, and *Pglyrp4*^-/-^ mice, of *Flavobacteria* in *Pglyrp1*^-/-^ and *Pglyrp3*^-/-^ mice, and of *Synechococcophycideae* and *Spartobacteria* in *Pglyrp3*^-/-^ and *Pglyrp4*^-/-^ mice.

We hypothesized that bacterial species that are more abundant in *Pglyrp*-deficient mice than in WT mice predispose *Pglyrp*-deficient mice to colitis, whereas other bacterial species that are more abundant in WT than in *Pglyrp*-deficient mice protect WT mice from colitis. We also hypothesized that an increase in the most abundant bacterial species may protect from colitis, whereas an increase in the species that are of low abundance may promote colitis, because we found statistically significant decrease in highly abundant species and OTUs and an increase in low abundant species and OTUs in all *Pglyrp*-deficient mice compared with WT mice (Fig 1B).

**Fig 3:**
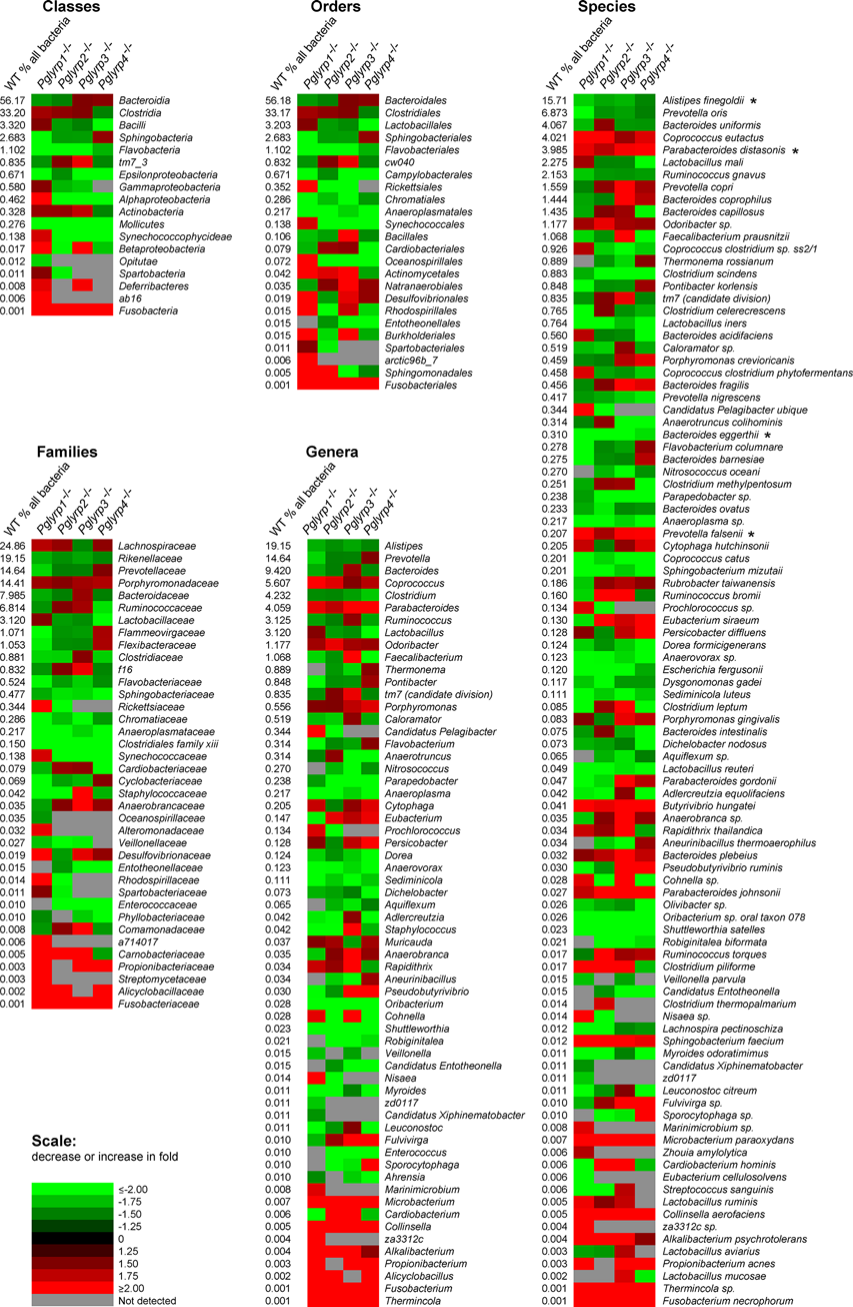
Bacteria with significantly changed abundance in stools of *Pglyrp*-deficient mice. Heatmap representation of the fold increase (red) or decrease (green) in the abundance of the indicated bacterial taxons, calculated as a ratio: % abundance in *Pglyrp*-deficient mice / % abundance in WT mice. The % abundance in WT mice is shown on the left of each heatmap panel. Taxons with no significant difference in abundance in any of the *Pglyrp*-deficient strains and low frequency taxons not detected in WT mice are omitted; * denotes species used in this study for colonizing mice. The % abundance of significantly changed microflora, *P* for abundance in *Pglyrp*-deficient *versus* WT microflora, and the ratio of abundance in *Pglyrp*-deficient to WT microflora for each strain and taxonomic unit are shown in S1–S6 Tables.

**Fig 4:**
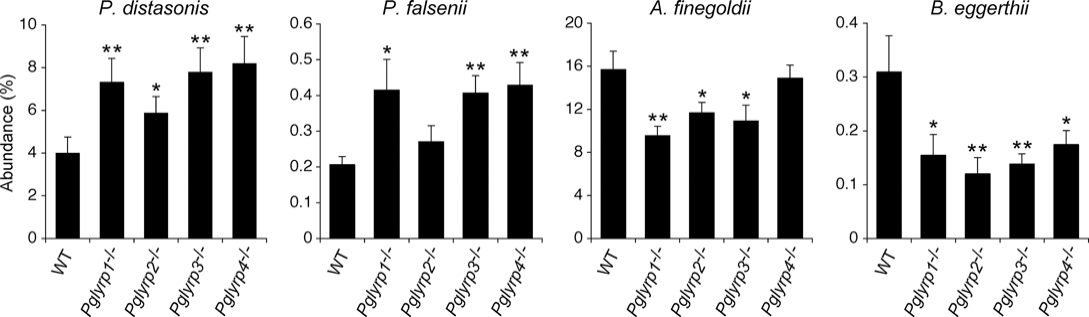
Abundance of *P*. *distasonis*, *P*. *falsenii*, *A*. *finegoldii*, and *B*. *eggerthii* in WT and *Pglyrp*-deficient mice. These species were selected for *in vivo* testing of their effect on susceptibility to colitis in Figs 5–8. The results are means ± SEM; *N* = 12 mice/strain; *, *P*<0.05; **, *P*<0.001 *Pglyrp*-deficient *versus* WT.

To identify bacterial species that predispose to or protect from DSS-induced colitis, in this study we focused on the common changes in microbiota, because we hypothesized that species consistently and significantly increased or decreased in all four *Pglyrp*-deficient strains of mice would be most likely responsible for their changed sensitivity to colitis. To test this hypothesis, we identified bacterial species with the most consistently increased or decreased abundance in all four *Pglyrp*-deficient strains of mice (with statistically significant change in at least three *Pglyrp*-deficient strains, including *Pglyrp3*^-/-^ mice, which are the most sensitive to colitis [13]). By these criteria all *Pglyrp*-deficient mice had increased abundance of two *Bacteroidetes* (*Parabacteroides distasonis* and *Prevotella falsenii*) (Figs 3 and 4 and S5 Table), and, therefore, we selected these two species for *in vivo* testing as candidate colitis-predisposing species.

Similar analysis revealed reduced abundance of three *Bacteroidetes* (*Alistipes finegoldii*, *Bacteroides eggerthii*, and *Parapedobacter sp*.), one *Firmicutes* (*Oribacterium sp*. *oral taxon 078*), one *Tenericutes* (*Anaeroplasma sp*.), one *Proteobacteria* (*Candidatus Entotheonella sp*.), and one *Actinobacteria* (*Microbacterium paraoxydans*) (Fig 3 and S5 Table). From this list of decreased species we selected two *Bacteroidetes* (*Alistipes finegoldii* and *Bacteroides eggerthii*) for *in vivo* testing (Fig 4), based on the following additional considerations: (i) one high and one lower abundance species to match the abundance of the selected proposed colitis-predisposing *Bacteroidetes* species, of which one has high and one low abundance; (ii) both species from *Bacteroidetes*, because changes in *Bacteroidetes* were previously linked to changes in sensitivity to colitis in immunodeficient mice, in contrast to changes in the species from other groups (*Firmicutes* and *Proteobacteria*), which were not previously linked to transmission of changed sensitivity to colitis in immunodeficient mice [21, 22]; (iii) presence of these species in humans; (iv) their availability from strain repositories; and (v) their ability to grow in culture.

### *Prevotella falsenii*, *Parabacteroides distasonis* and *Bacteroides eggerthii* enhance and *Alistipes finegoldii* attenuates colitis in mice

Here we tested the hypothesis that individual bacterial species (*P*. *falsenii*, *P*. *distasonis*, *B*. *eggerthii*, and *A*. *finegoldii*), selected as described in the preceding section based on their increased or decreased abundance in the stools of all *Pglyrp*-deficient mice, predispose to or protect from DSS-induced colitis in mice.

First we used a well-established model with WT mice depleted of their intestinal microflora by a 3-week long treatment with oral ciprofloxacin and metronidazole [21, 23]. We selected this model rather than germ-free mice (which are often used in such experiments), because the innate and adaptive immune systems of germ-free mice are immature and for that reason colonization of germ-free mice with bacteria may sometimes produce different effects than in conventional animals [24, 25]. Previously in this antibiotic depletion model colitogenic members of normal flora bacteria induced severe colitis only in genetically-susceptible immunodeficient mice, but not WT mice [21].

**Fig 5:**
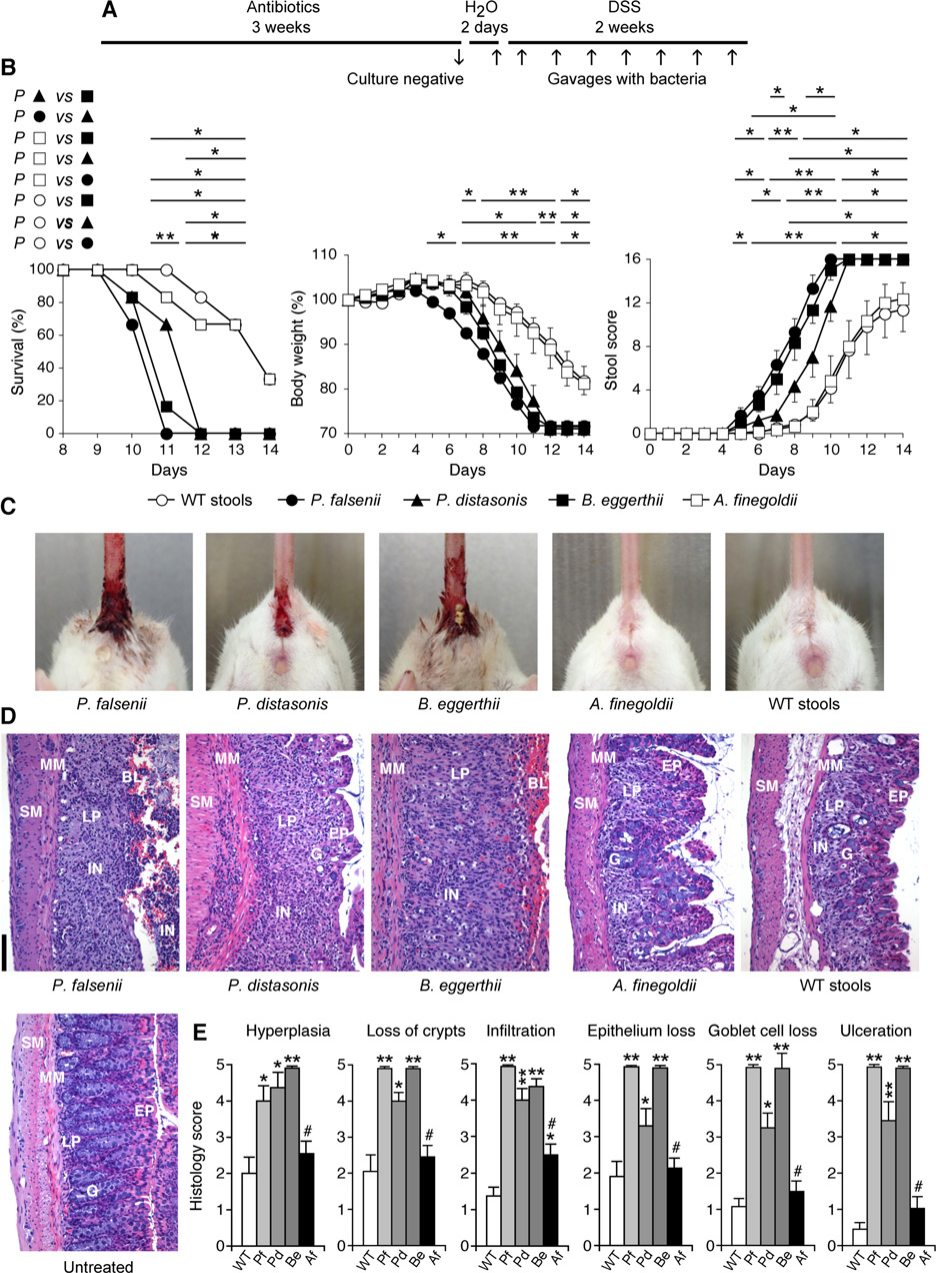
*P*. *falsenii*, *P*. *distasonis*, and *B*. *eggerthii*, but not *A*. *finegoldii*, predispose mice depleted of intestinal microflora to colitis. (**A**) WT mice were depleted of intestinal microflora by treatment with antibiotics and then gavaged every other day with *P*. *falsenii*, or *P*. *distasonis*, or *B*. *eggerthii*, or *A*. *finegoldii*, or stools from WT mice as a control, and also treated with DSS. (**B**) Mice were monitored over time for survival, change in body weight, and stool scores and rectal bleeding. (**C**) Gross rectal bleeding on day 10 in mice gavaged with *P*. *falsenii*, or *P*. *distasonis*, or *B*. *eggerthii*, but not in mice gavaged with *A*. *finegoldii* or stools from WT mice. (**D**) Representative hematoxylin-eosin stained sections from day 9 of the colons from mice gavaged with bacteria or WT stools or from untreated mice as indicated; BL, blood; EP, epithelial cells; LP, lamina propria; G, goblet cells; MM, muscularis mucosa; SM, submucosa; and IN, inflammatory cell infiltrations are indicated; size bar = 100 μm. (**E**) Severity of histopathological changes in the colon of mice gavaged with *P*. *falsenii* (Pf), or *P*. *distasonis* (Pd), or *B*. *eggerthii* (Be), or *A*. *finegoldii* (Af), or stools from WT mice and treated with DSS. The results are means ± SEM of 6 (in B) or 4 (in E) mice/group; significance of differences: *, *P*<0.05; **, *P*<0.001; as indicated (in B) or *versus* WT (in E); #, *P*<0.05 Af *versus* Pf, Pd, and Be (all *P*<0.001 for Af *versus* Pf and Be, except for hyperplasia).

We grew representative type strains (obtained from ATCC or Riken) of our candidate bacterial species *in vitro* and we colonized microflora-depleted WT mice by multiple gavages with *P*. *falsenii*, or *P*. *distasonis*, or *B*. *eggerthii*, or *A*. *finegoldii*, or with total preserved WT microflora (as a control), and we induced colitis by treatment with oral DSS (Fig 5A).

Mice gavaged with *P*. *falsenii*, or *P*. *distasonis*, or *B*. *eggerthii* developed much more severe DSS-induced colitis than control mice gavaged with the entire WT microflora, manifested by significantly shortened survival, significantly accelerated loss of body weight, significantly accelerated and increased stool scores and intestinal bleeding (Fig 5B), and frank bleeding from the anus (Fig 5C). We also evaluated histopathological changes as a measure of the severity of colitis. Mice gavaged with *P*. *falsenii*, or *P*. *distasonis*, or *B*. *eggerthii* had significantly more pronounced hyperplasia of lamina propria, loss of crypts, infiltration with inflammatory cells, loss of epithelium, loss of goblet cells, and ulceration, compared with mice gavaged with WT stools, which after the same duration of DSS treatment had only low histopathological changes (Fig 5D and 5E). The colitis was most severe in mice gavaged with *P*. *falsenii* or *B*. *eggerthii*, and moderately severe with *P*. *distasonis*. By contrast, based on the same criteria, mice gavaged with *A*. *finegoldii* showed significantly less severe colitis than mice gavaged with *P*. *falsenii*, or *P*. *distasonis*, or *B*. *eggerthii*. Colitis in mice gavaged with *A*. *finegoldii* was similar in severity and not significantly different than colitis in mice gavaged with WT stools, except for somewhat more severe colon infiltration with inflammatory cells (Fig 5B–5D).

We next evaluated possible synergistic effect of gavaging microflora-depleted mice with two bacterial species, because these bacterial species exist together in the intestine, because the abundance of both *P*. *falsenii* and *P*. *distasonis* was increased and the abundance of both *B*. *eggerthii* and *A*. *finegoldii* was decreased in *Pglyrp*-deficient mice, and also because monoassociation with single bacterial species in some studies was not sufficient to affect the sensitivity to colitis [26].

**Fig 6:**
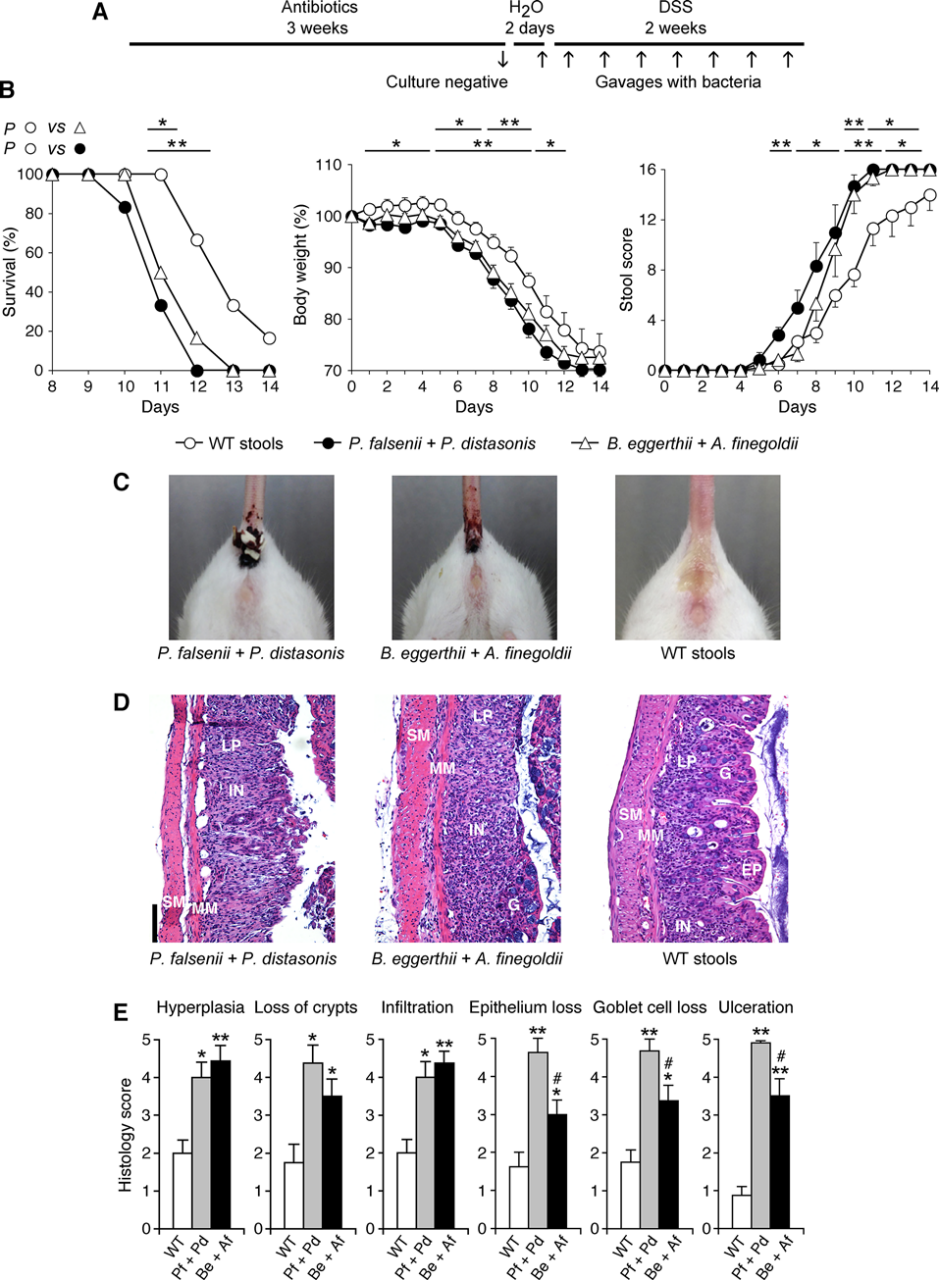
*P*. *falsenii* plus *P*. *distasonis*, and *B*. *eggerthii* plus *A*. *finegoldii* predispose mice depleted of intestinal microflora to colitis. (**A**) WT mice were depleted of intestinal microflora by treatment with antibiotics and then gavaged every other day with *P*. *falsenii* plus *P*. *distasonis*, or *B*. *eggerthii* plus *A*. *finegoldii*, or with stools from WT mice as a control, and also treated with DSS. (**B**) Mice were monitored over time for survival, change in body weight, and stool scores and rectal bleeding. (**C**) Gross rectal bleeding on day 10 in mice gavaged with *P*. *falsenii* plus *P*. *distasonis*, or *B*. *eggerthii* plus *A*. *finegoldii*, but not in mice gavaged with stools from WT mice. (**D**) Representative hematoxylin-eosin stained sections from day 9 of the colons from mice gavaged with bacteria or WT stools as indicated; EP, epithelial cells; LP, lamina propria; G, goblet cells; MM, muscularis mucosa; SM, submucosa; and IN, inflammatory cell infiltrations are indicated; size bar = 100 μm. (**E**) Severity of histopathological changes in the colon of mice gavaged with *P*. *falsenii* plus *P*. *distasonis* (Pf + Pd), or *B*. *eggerthii* plus *A*. *finegoldii* (Be + Af), or stools from WT mice and treated with DSS. The results are means ± SEM of 6 (in B) or 4 (in E) mice/group; significance of differences: *, *P*<0.05; **, *P*<0.001; as indicated (in B) or *versus* WT (in E); #, *P*<0.05 Be + Af *versus* Pf + Pd.

Mice gavaged with *P*. *falsenii* plus *P*. *distasonis* also developed more severe DSS-induced colitis than control mice gavaged with the entire WT microflora, manifested by significantly shortened survival, significantly accelerated loss of body weight, significantly accelerated and increased stool scores and intestinal bleeding (Fig 6B), and frank bleeding from the anus (Fig 6C). Mice gavaged with *P*. *falsenii* plus *P*. *distasonis* also had significantly more pronounced hyperplasia of lamina propria, loss of crypts, infiltration with inflammatory cells, loss of epithelium, loss of goblet cells, and ulceration, compared with mice gavaged with WT stools, which after the same duration of DSS treatment had only moderate infiltration with inflammatory cells and very few other histopathological changes (Fig 6D and 6E). However, the severities of colitis in mice gavaged with two species (*P*. *falsenii* plus *P*. *distasonis*, Fig 6) and single species (*P*. *falsenii* or *P*. *distasonis*, Fig 5) were similar. These results indicate that gavaging these two colitis-promoting bacterial species together does not further enhance the sensitivity to DSS-induced colitis, compared with gavaging single species.

Mice gavaged with *B*. *eggerthii* plus *A*. *finegoldii* also developed more severe DSS-induced colitis than mice gavaged with the entire WT microflora (Fig 6B–6E), although less severe than mice gavaged with *P*. *falsenii* or *B*. *eggerthii* individually (Fig 5B–5E), or with *P*. *falsenii* plus *P*. *distasonis* (Fig 6B–6E). These results indicate that *A*. *finegoldii* is a colitis-protective species, because it attenuates the severity of DSS-induced colitis promoted by *B*. *eggerthii*.

**Fig 7:**
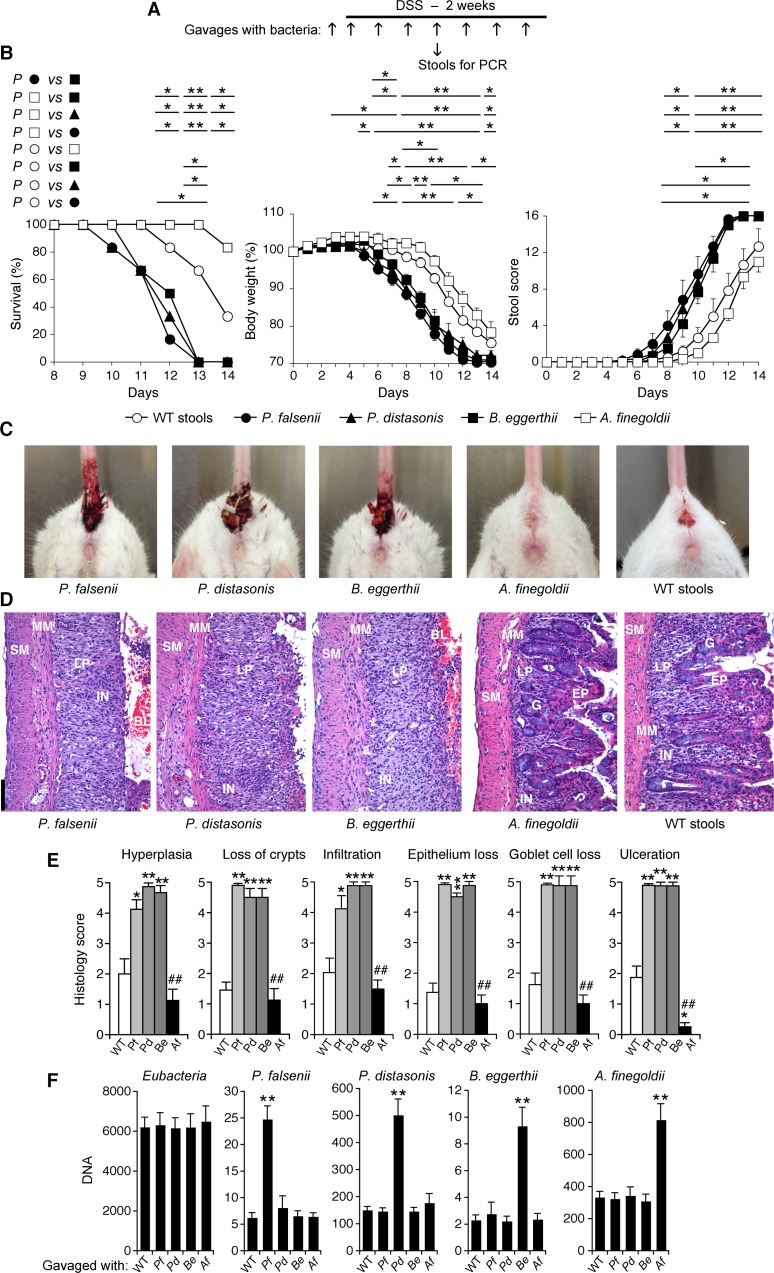
*P*. *falsenii*, *P*. *distasonis*, and *B*. *eggerthii* predispose WT mice with intact intestinal microflora to colitis, whereas *A*. *finegoldii* protects WT mice with intact intestinal microflora from colitis. (**A**) WT mice were gavaged every other day with *P*. *falsenii*, or *P*. *distasonis*, or *B*. *eggerthii*, or *A*. *finegoldii*, or stools from WT mice as a control, and also treated with DSS. (**B**) Mice were monitored over time for survival, change in body weight, and stool scores and rectal bleeding. (**C**) Severe gross rectal bleeding on day 12 in mice gavaged with *P*. *falsenii*, or *P*. *distasonis*, or *B*. *eggerthii*, mild bleeding in mice gavaged with stools from WT mice, and not in mice gavaged with *A*. *finegoldii*. (**D**) Representative hematoxylin-eosin stained sections from day 11 of the colons from mice gavaged with bacteria or WT stools as indicated; BL, blood; EP, epithelial cells; LP, lamina propria; G, goblet cells; MM, muscularis mucosa; SM, submucosa; and IN, inflammatory cell infiltrations are indicated; size bar = 100 μm. (**E**) Severity of histopathological changes in the colon of mice gavaged with *P*. *falsenii* (Pf), or *P*. *distasonis* (Pd), or *B*. *eggerthii* (Be), or *A*. *finegoldii* (Af), or stools from WT mice and treated with DSS. (**F**) Abundance of DNA for the indicated bacteria in stools on day 9. The results are means ± SEM of 6 (in B and F) or 4 (in E) mice/group; significance of differences: *, *P*<0.05; **, *P*<0.001; as indicated (in B) or *versus* WT (in E and F); ##, *P*<0.001 Af *versus* Pf, Pd, and Be.

**Fig 8:**
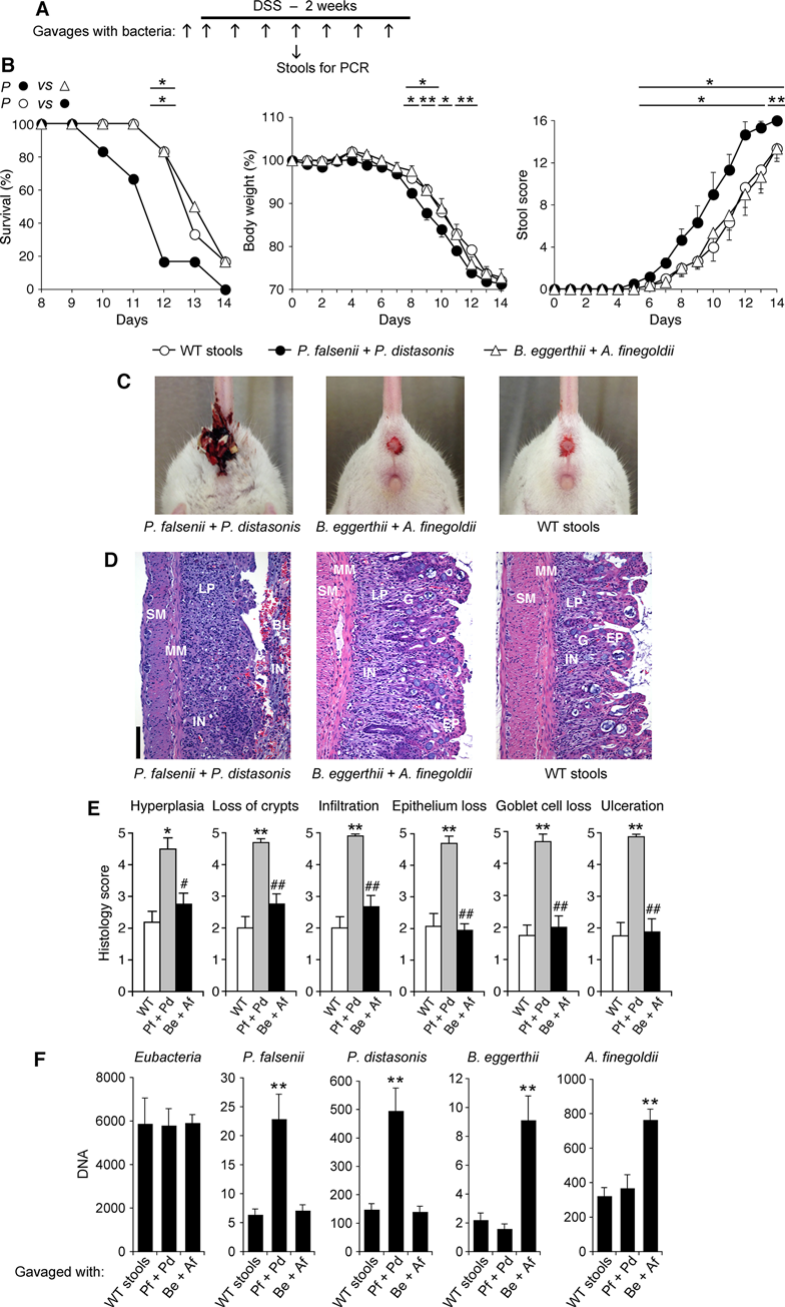
*P*. *falsenii* plus *P*. *distasonis*, but not *B*. *eggerthii* plus *A*. *finegoldii*, predispose WT mice with intact intestinal microflora to colitis. (**A**) WT mice were gavaged every other day with *P*. *distasonis* plus *P*. *falsenii*, or *A*. *finegoldii* plus *B*. *eggerthii*, or with stools from WT mice as a control, and also treated with DSS. (**B**) Mice were monitored over time for survival, change in body weight, and stool scores and rectal bleeding. (**C**) Severe gross rectal bleeding on day 12 in mice gavaged with *P*. *falsenii* plus *P*. *distasonis*, and mild bleeding in mice gavaged with *B*. *eggerthii* plus *A*. *finegoldii*, or with stools from WT mice. (**D**) Representative hematoxylin-eosin stained sections from day 11 of the colons from mice gavaged with bacteria or WT stools as indicated; BL, blood; EP, epithelial cells; LP, lamina propria; G, goblet cells; MM, muscularis mucosa; SM, submucosa; and IN, inflammatory cell infiltrations are indicated; size bar = 100 μm. (**E**) Severity of histopathological changes in the colon of mice gavaged with *P*. *falsenii* plus *P*. *distasonis* (Pf + Pd), or *B*. *eggerthii* plus *A*. *finegoldii* (Be + Af), or stools from WT mice and treated with DSS. (**F**) Abundance of DNA for the indicated bacteria in stools on day 9. The results are means ± SEM of 6 (in B and F) or 4 (in E) mice/group; significance of differences: *, *P*<0.05; **, *P*<0.001; as indicated (in B) or *versus* WT (in E and F); #, *P*<0.05; ##, *P*<0.001; Be + Af *versus* Pf + Pd.

We next tested the role of intact WT microflora in protection against DSS-induced colitis to answer two questions: (i) whether our proposed colitis-promoting bacteria would still promote colitis if the remaining WT microflora were present; and (ii) whether potentially protective microflora requires for manifestation of its protection the presence of the remaining intact WT microflora. To answer these two questions, we gavaged unmanipulated WT mice, whose intestinal microflora was intact, with *P*. *falsenii*, or *P*. *distasonis*, or *B*. *eggerthii*, or *A*. *finegoldii*, or with *P*. *falsenii* plus *P*. *distasonis*, or with *B*. *eggerthii* plus *A*. *finegoldii*, every other day, to increase the abundance of these bacteria in the intestine. We then tested their sensitivity to DSS-induced colitis (Figs 7A and 8A).

WT mice gavaged every other day with *P*. *falsenii*, or *P*. *distasonis*, or *B*. *eggerthii*, or with *P*. *falsenii* plus *P*. *distasonis* and treated with DSS developed more severe colitis than control mice gavaged with the entire WT microflora, manifested by significantly shortened survival, significantly accelerated loss of body weight, and significantly accelerated and increased stool scores and intestinal bleeding (Figs 7B and 8B), and also severe frank bleeding from the anus (Figs 7C and 8C). These mice also had significantly more pronounced hyperplasia of lamina propria, loss of crypts, infiltration with inflammatory cells, loss of epithelium, loss of goblet cells, and ulceration, compared with mice gavaged with WT stools, which after the same duration of DSS treatment had only moderate histopathological changes (Figs 7D and 7E and 8D and 8E). Thus, these results indicate that *P*. *falsenii*, *P*. *distasonis*, and *B*. *eggerthii* increase the sensitivity of WT mice to DSS-induced colitis not only when they are present by themselves when the remaining microflora is depleted (Figs 5 and 6), but also when their abundance is increased and the remaining intestinal microflora is intact (Figs 7 and 8).

By contrast, WT mice with intact microflora gavaged with *A*. *finegoldii* were more resistant to DSS-induced colitis than mice gavaged with WT bacteria, with no frank bleeding from the anus (Fig 7C), significantly lower loss of body weight (on days 8–10) (Fig 7B), and significantly less colon ulceration (Fig 7D and 7E). WT mice with intact microflora gavaged with *B*. *eggerthii* plus *A*. *finegoldii* had similar sensitivity to DSS-induced colitis as mice gavaged with WT bacteria, based on similar survival, loss of body weight, stool scores, anal bleeding, and histopathological changes in the colon (Fig 8B–8E), which was significantly less severe than the DSS-induced colitis in mice with intact flora gavaged just with *B*. *eggerthii* (Fig 7). Thus, increased abundance of *A*. *finegoldii* had a protective effect against DSS-induced colitis both in WT mice with otherwise unmanipulated normal microflora, as well as against increased sensitivity to DSS-induced colitis caused by higher abundance of a colitis-promoting species *B*. *eggerthii*. We confirmed by qPCR of bacterial DNA that these bacterial gavages actually selectively increased the abundance of the gavaged bacteria in the stools (Figs 7F and 8F).

Our results also show that the presence of WT intact intestinal microflora protects against increased sensitivity to DSS-induced colitis caused by higher abundance of *P*. *falsenii*, *P*. *distasonis*, or *B*. *eggerthii*, because the mortality, weight loss, stool scores, intestinal bleeding, frank bleeding from the anus, and histopathological changes in mice with intact intestinal microflora were delayed by approximately 2 days, when compared with mice with antibiotic-depleted microflora, and this delay was statistically significant at *P*<0.05 (compare Figs 5 and 6, in which pictures of bleeding from the anus were taken on day 10 and colons for histopathology were resected on day 9, with Figs 7 and 8, in which pictures were taken on day 12 and colons were resected on day 11).

## Discussion

Dysbiosis is a well-recognized hallmark of IBD, which is usually associated with a decreased diversity of intestinal microflora, lower abundance of *Firmicutes*, an increase or a decrease in certain members of *Bacteroidetes*, and often an increase in some *Proteobacteria* [27–35]. Accordingly, fecal transplantation is used to treat IBD, but the success of this treatment is inconsistent, most likely because of the incomplete knowledge which specific bacteria protect from and which predispose to IBD in the context of all other variables that affect the outcome of IBD, such as the host genotype and the composition of the entire donor and recipient microflora [36–38].

To define the causative role of various members of the intestinal microbiota in colitis, several models of genetically-determined and/or environmentally-induced IBD have been used [39]. From these studies, the disease-promoting or disease-protecting role of intestinal microbiome in animal models of IBD is well established. Various immunodeficient mice have changed microbiota and their increased sensitivity to colitis can be transferred to genetically non-susceptible hosts with intestinal microflora [13, 14, 21, 22, 40–43]. There are also some examples of pathobionts, which are benign commensals or beneficial symbionts in WT but opportunistic pathogens in genetically-susceptible immunocompromised individuals. These pathobionts include *Helicobacter hepaticus* in *IL10*^-/-^ mice [44–46], *Escherichia coli* in *TLR5*^-/-^ mice [47], and *Bacteroides thetaiotaomicron* in *IL10r2*^-/-^*Tgfbr2*^-/-^ mice [21]. But even the susceptibility to these pathobionts in immunodeficient mice is dependent both on the host genotype and the composition of its microbiome [48].

However, the specific changes in the intestinal microbiome responsible for the transfer of increased sensitivity to IBD to genetically colitis-resistant WT mice are still not clear. There are only some examples of individual bacteria or their products conferring increased resistance to colitis in WT mice, most notably *Faecalibacterium prausnitzii* [49], multiple *Clostridium* species [50], and *Bacteroides fragilis* and its polysaccharide A [51, 52]. These bacteria are typically decreased in IBD patients [30].

The exact knowledge of colitis-promoting and colitis-protective bacteria that are active in hosts with various genetic backgrounds, including WT hosts, would greatly aid in rational designing of microbiota-based prevention and treatment of IBD aimed at optimal balancing of intestinal microbiota. Towards this goal, we have previously shown that dysbiotic microflora from *Pglyrp*-deficient mice is sufficient to increase sensitivity to DSS-induced colitis irrespective of genotype [13, 14]. Here we characterized in detail this dysbiotic microflora and identified both common and unique changes in intestinal microbiota in all four *Pglyrp*-deficient strains.

The most significant common changes in β-diversity in all *Pglyrp*-deficient mice compared with WT mice were: (i) decrease in high abundant and increase in low abundant species and OTUs; (ii) increased abundance of two *Bacteroidetes* (*P*. *falsenii* and *P*. *distasonis*); and (iii) reduced abundance of three *Bacteroidetes* (*A*. *finegoldii*, *B*. *eggerthii*, and *Parapedobacter sp*.) and four other species, each belonging to a different taxon. From these species we selected four members of *Bacteroidales*: *P*. *distasonis*, *P*. *falsenii*, *A*. *finegoldii*, and *B*. *eggerthii* as candidate colitis-promoting or colitis-protecting species for *in vivo* testing of their effect on sensitivity to DSS-induced colitis. The abundance of *Bacteroidales* is often changed in IBD patients, as members of this order may be increased or decreased in IBD, and they may have colitis predisposing or protective effects [13, 22, 26, 28, 29, 42, 51, 53–58].

*P*. *falsenii*, *P*. *distasonis*, and *B*. *eggerthii* increased sensitivity to DSS-induced colitis in both WT mice depleted of their own intestinal flora with antibiotics, and in WT mice with intact intestinal flora, which is a more realistic scenario resembling the situation in *Pglyrp*-deficient mice or IBD patients. These results identify *P*. *falsenii*, *P*. *distasonis*, and *B*. *eggerthii* as colitis promoting species and indicate that they are at least partially responsible for the increased sensitivity of *Pglyrp*-deficient mice to DSS-induced colitis. The results with *P*. *falsenii* and *P*. *distasonis* support our initial hypothesis that these two species more abundant in *Pglyrp*-deficient mice predispose to colitis. However, the results with *B*. *eggerthii*, which also predisposes to colitis, indicate that some species with decreased abundance in *Pglyrp*-deficient mice may be also able to promote colitis if their abundance is increased. These results suggest that increasing the abundance of normally low abundant species (*P*. *falsenii* and *B*. *eggerthii*) greatly enhances the sensitivity to colitis. Of note, these bacteria do not cause colitis on their own, but rather they predispose to colitis induced by DSS, which is an intestinal epithelium-damaging compound. Moreover, our results do not exclude the possibility that changes in the abundance of other bacterial species could also affect the sensitivity to colitis.

Our results are consistent with the previous studies showing correlation between the increase in the abundance of *Prevotellaceae* and the severity of microflora-transmissible colitis in *NLRP6* inflammasome-deficient mice [22], in *IL22*^-/-^ mice [42], and in *Casp3/11*^-/-^ mice [53]. *Prevotellaceae* produce sulfatases that degrade mucus polysaccharide, which could disrupt intestinal mucosal barrier [59], and an increase in sulfatases was found in biopsies from IBD patients [60]. Moreover, the abundance of *Prevotellaceae* is increased in the colon [54] and in the oral cavity [61] of IBD patients, and is also implicated in the pathogenesis of periodontal disease [62]. *Pglyrp3* and *Pglyrp4* have the highest expression in the oral cavity and esophagus [5, 6]. Because Pglyrps are secreted antibacterial proteins [7–11], they likely play a role in maintaining microflora in the oral cavity and in shaping the composition of ingested bacteria that pass through the mouth and esophagus to colonize the remaining parts of the intestinal tract.

There are conflicting reports on the role of *P*. *distasonis* and *Bacteroides* in IBD. The abundance of *Parabacteroides sp*. was increased in stools of colitis-prone *NHE3*^-/-^ mice [55], but was decreased in colitis-prone *IL22*^-/-^ mice [42], and was decreased in biopsies from inflamed ileum of pediatric patients with Crohn’s Disease (CD) and ulcerative colitis (UC) [56] (two main forms of IBD), and was increased during remission in ileal biopsies from IBD patients, along with increased abundance of *Prevotella* and *Bacteroides* [57]. *P*. *distasonis* (formerly *Bacteroides distasonis*) was also decreased in washed tissue sections from small or large intestine of CD and UC patients, representing epithelium-associated bacteria [29]. Furthermore, oral administration of membrane fractions from *P*. *distasonis* attenuated DSS-induced colitis in mice [63]. These discrepant results may be due to differences between animal models and patients and different sources of samples, as some assays (including ours) were performed on stools and represent luminal distal colon and fecal bacteria, whereas in several patient studies tissue-associated bacteria from more proximal segments of intestine were used, and colon and ileum often show opposite changes in the abundance and types of bacteria [32].

Although we are not aware of any previous reports linking *B*. *eggerthii* with increased sensitivity to colitis, some *Bacteroides* species have increased abundance [54] and some decreased abundance [29, 57, 58] in IBD. Also, *Bacteroides* were proposed to have beneficial protective effect against IBD, because they break down complex dietary carbohydrates, modulate mucosal glycosylation, and promote angiogenesis and immune maturation [52, 64, 65]. Some *Bacteroides* also produce a colitis-protective polysaccharide [51]. Moreover, *B*. *eggerthii* is one of the intestinal bacteria that generate phenolic acids, which are regarded as beneficial for human health [66]. However, our results are not consistent with these hypotheses and show that increased abundance of *B*. *eggerthii* promoted DSS-induced colitis in WT mice with otherwise intact normal microflora and also with antibiotic-depleted microflora.

*A*. *finegoldii*, whose abundance was decreased in colitis-sensitive *Pglyrp*-deficient mice, attenuated DSS-induced colitis when its abundance was increased in WT mice with intact intestinal microflora or when it was administered together with a colitis-predisposing bacterium, *B*. *eggerthii*. These results are consistent with decreased abundance of *Alistipes sp*. in IBD [29]. The entire normal microflora also had colitis protective effect, which could be replicated with *A*. *finegoldii*, the most abundant bacterial species in WT mice. Thus, the colitis-protective properties of *A*. *finegoldii* may be the reason for its highest abundance in the colon.

In addition to the increased sensitivity to colitis [13, 14], *Pglyrp*-deficient mice also have changed sensitivity to other inflammatory diseases. *Pglyrp2* protects mice against psoriasis-like skin inflammation [16], is required for the development of experimental arthritis [15], and exacerbates bacterial keratitis [19]. *Pglyrp3* and *Pglyrp4* protect mice against atopic dermatitis [17]. *Pglyrp1* has a pro-inflammatory effect in three mouse models of inflammatory skin diseases (psoriasis, atopic dermatitis, and contact dermatitis) [16, 17] and in experimentally induced asthma [18], but has anti-inflammatory effect in experimentally induced arthritis [15]. Thus, in contrast to the similar effect on the sensitivity to colitis, each *Pglyrp* has a unique role in the development of other inflammatory diseases, which is consistent with *Pglyrps* not compensating for each other in mice deficient in a single *Pglyrp*. It is not known whether the above-mentioned changed sensitivities to non-intestinal inflammatory diseases are also based on the changes in microflora. If they are, the unique changes in the microbiomes of *Pglyrp*-deficient mice would be likely involved, which will be the subject of our future studies.

Several lines of evidence suggest that our results on these mouse models are relevant to human diseases. We have demonstrated genetic association of 16 human *Pglyrp* variants in IBD patients with CD and UC [67]. Human *Pglyrp2* gene is located in the IBD susceptibility locus 19p13 [68] and human *Pglyrp3* and *Pglyrp4* genes are located at 151.5 Mb on chromosome 1 near the 151.79 Mb IBD locus [69]. Also, human *Pglyrp3* and *Pglyrp4* are genetically associated with psoriasis [70, 71].

In conclusion, our results show that increased abundance of *P*. *falsenii*, *P*. *distasonis*, or *B*. *eggerthii* enhances DSS-induced colitis in both WT mice with otherwise undisturbed intestinal microflora and in WT mice with antibiotic-depleted intestinal microflora. By contrast, increased abundance of *A*. *finegoldii* (which is the most abundant species in WT mice) attenuates DSS-induced colitis both in WT mice with otherwise undisturbed intestinal microflora and in WT mice with antibiotic-depleted intestinal microflora, similar to the colitis protective effect of the entire normal flora. These results identify *P*. *falsenii*, *P*. *distasonis*, and *B*. *eggerthii* as colitis-promoting species and *A*. *finegoldii* as colitis-protective species.

## Materials and Methods

### Mice and ethics statement

*Pglyrp1*^-/-^, *Pglyrp2*^-/-^, *Pglyrp3*^-/-^, and *Pglyrp4*^-/-^ mice were described previously [13, 15, 72]. All knockout and WT mice were on BALB/c background, female, 7–9 week-old, bred and kept under conventional pathogen-free conditions in the same room in our facility to minimize the influence of differences in the environment. The original colony founder BALB/c mice for both WT and *Pglyrp*-deficient mice were obtained from Harlan-Sprague-Dawley. For each experiment, mice from several different cages and breeder pairs were used. We did not use WT and homozygous knockout littermates from heterozygous breeding pairs because this strategy may skew the results to the particular microflora present only in this breeding pair; and also because the effect of *Pglyrps* on the composition of the microbiome is not instantaneous, but takes time, and stabilization of microbiome characteristic of a given mutant strain takes more than one generation. To avoid changes in microbiome that could accumulate over extended period of time, we backcross our mutant mice to WT females once every other year and re-derive our homozygous knockout breeding pairs. The latter strategy also minimizes genetic drift in the population. The BALB/c background of knockout mice and their negative status for all common viral and bacterial pathogens and parasites (including negative PCR stool tests for mouse Norovirus) were confirmed as previously described [13, 14, 18].

All experiments on mice were performed according to the guidelines and approved by the Indiana University School of Medicine–Northwest Institutional Animal Care and Use Committee (approval number IUSM-NW-IACUC-16). All efforts were made to minimize suffering of animals.

### Stool collection and DNA extraction

We collected and immediately snap-froze at -80°C freshly defecated stools from individual 8-week-old female WT, *Pglyrp1*^-/-^, *Pglyrp2*^-/-^, *Pglyrp3*^-/-^, and *Pglyrp4*^-/-^ mice (200 mg/mouse, 12 mice/strain) and isolated DNA from microflora using Qiagen QIAamp DNA Stool Mini Kit. For each strain, mice originated from six different litters from different parents (2 mice per litter), weaned into separate cages, and all cages were kept in the same room in our animal facility. This strategy allows stabilization of microflora and minimizes the variability observed between different litters due to different parents and different cages and rooms [13, 14].

### Pyrosequencing of 16S rRNA genes and microbiome analysis

We performed genetic phylotyping (community profiling) using pyrosequencing of the variable regions of bacterial 16S ribosomal RNA (rRNA) genes using Roche 454 titanium technology. Sequencing data were processed, including depletion of barcodes and primers, removal of short sequences (<150 bp), sequences with ambiguous base calls, and sequences with homopolymer runs exceeding 6 bp, de-noising, and removal of chimeras [73, 74]. Operational taxonomic units (OTUs) were then defined after removal of singleton sequences and clustering, and taxonomically classified using BLASTn against a curated GreenGenes database and assigned to species at >97% of identity to the reference sequence (<3% divergence), to unclassified species at 95%–97% identity, genus at 90%–95% identity, family at 85%–90% identity, order at 80%-85% identity, phylum at 77%-80% identity, and unclassified at <77% identity [75]. We compared the sequences and analyzed the changes in bacterial ecology using Quantitative Insights Into Microbial Ecology (QIIME; http://qiime.sourceforge.net), Bayesian, PyNAST, and UniFrac [76–78]. The Shannon diversity index H = –Σ*p*_*i*_ ln(*p*_*i*_) and Shannon equitability index E_H_ = H/ln(S), where *p*_*i*_ is the proportion of the *i*th taxonomic unit (OTU or species) and S is the total number of taxonomic units, were calculated using Microsoft Excel. The average total number of reads per mouse was 7354, with no significant differences between the numbers of reads/mouse between WT and *Pglyrp*-deficient mice.

### Bacteria

*Parabacteroides distasonis* (ATCC 8503), *Prevotella falsenii* (15124, Riken Bioresource Center, Japan), *Alistipes finegoldii* (16770, Riken Bioresource Center, Japan), and *Bacteroides eggerthii* (12986, Riken Bioresource Center, Japan), as well as stool cultures, were grown in liquid EG Medium or Modified Pre-Reduced Clostridial Broth (*P*. *distasonis*), or on pre-reduced agar plates with the same media plus 5% horse blood under anaerobic conditions in complete absence of oxygen (90% N_2_, 5% H_2_, 5% CO_2_) in Anaerobe Systems AS-580 Anaerobic Chamber at 37°C. Aliquots of bacteria in deoxygenated Dulbecco’s PBS with 0.15% thioglycollate, 0.05% cysteine, and 15% glycerol were frozen at -80°C and thawed out once for gavaging.

### Colitis model

Experimental colitis was induced in mice with 5% DSS (dextran sulfate sodium, MP Biomedical) in drinking water [13, 14, 39]. DSS-induced intestinal inflammation is a well-established animal model for colitis and its manifestations include bloody diarrhea, weight loss, shortening of the colon, mucosal ulceration, epithelial dysplasia, and intestinal bleeding. Manifestations such as predominant left-sided colitis, epithelial dysplasia and lack of granulomas are similar to UC, although the complexity of the human disease is not completely reproduced in the DSS model [39]. The development and severity of colitis was evaluated as previously described [13], using: (a) mortality; (b) weight loss calculated as % of initial body weight; and (c) stool and rectal bleeding scores on a scale of 0–16, graded as follows: (i) stool consistency: 0, normal; 2, pasty and semi-formed stools that do not adhere to the anus; 4, liquid stools (may adhere to the anus); (ii) stool hemoccult assay: 0, negative test; 1, weakly positive test; 2, strongly positive test but no visible blood; 4, blood visible in the stools; and (iii) rectal bleeding: 4, frank bleeding from the anus and/or fresh or dried blood around the anus; 8, severe bleeding from the anus. The scores were added for each mouse with the maximum score of 16. For evaluation of histopathology, mice treated with 5% DSS were sacrificed on day 9 or 11, distal sections of the colon were fixed in 10% buffered formalin, embedded in paraffin, sectioned, stained with hematoxylin and eosin, and analyzed for hyperplasia of lamina propria, loss of crypts, infiltration with immune cells, loss of epithelium, loss of goblet cells, and extent of ulceration, to evaluate the severity of colitis. The scoring scale was from 0 to 5, with 0 being no change and 5 being the greatest change [14].

### Depletion of microflora and bacterial colonization by gavaging

To determine which gut microflora predisposes to or protects from colitis, two models were used. First, we used an established non-germ-free model of WT mice depleted of their intestinal microflora by a 3-week treatment with antibiotics in drinking water (containing 100 μg/ml ciprofloxacin, 250 μg/ml metronidazole, and 20 mg/ml Kool-Aid mix) [14, 21, 23]. Anaerobic stool cultures at the end of antibiotic treatment were negative (<10^3^ bacteria/g of feces, freshly collected into a reducing buffer). Microflora-depleted mice were then given sterile drinking water for 2 days, followed by gavaging into the stomach with WT stools (complete WT microbiota control) or selected cultured bacteria, followed by 5% DSS in sterile drinking water.

As the second model, we used untreated WT mice, which we gavaged into the stomach with cultured bacteria or WT stools (as a control) every other day and treated with 5% DSS in sterile drinking water, which was started one day after the first gavage. Just before the 5^th^ gavage, we took stool samples from all mice for DNA extraction and quantification of DNA of the gavaged bacteria, to confirm that the gavages indeed increased the abundance of the gavaged species.

To prepare stools for gavaging, 10 freshly defecated pellets (150 mg/mouse) were collected and pooled from 12 female 8-week-old WT mice originated from six different breeding parents and kept in separate cages after weaning in the same room in our animal facility. This strategy minimizes the variability observed between different litters due to parent-to-parent and cage-to-cage differences. Stools were immediately placed in a reduced preserving buffer (Dulbecco’s PBS with 0.15% thioglycollate, 0.05% cysteine, 15% glycerol) on ice to maintain the original composition of live bacteria, including anaerobes, homogenized with Polytrone, and filtered through 100 μm nylon cell strainer (BD Falcon) to remove debris [13]. The concentration of the stool suspension was adjusted based on OD_660_, and stool aliquots were kept at -80°C and thawed out once.

Mice were gavaged into the stomach every other day with 0.2 ml containing 6 mg of stools from WT mice (3x10^9^ bacteria) or 2x10^8^ cultured single bacterial species (*P*. *distasonis*, or *P*. *falsenii*, or *A*. *finegoldii*, or *B*. *eggerthii*), or 3x10^8^ cultured bacteria, containing 2:1 mixture of either *P*. *distasonis* plus *P*. *falsenii* or *A*. *finegoldii* plus *B*. *eggerthii*, for the entire duration of the experiment.

### Stool flora analysis by qPCR

Freshly defecated feces (100 mg/mouse) were collected from each mouse and immediately snap-frozen at -80°C. DNA was isolated from stools from each mouse using Qiagen QIAamp DNA Stool Mini Kit. The abundance of gavaged bacteria in mouse stools was measured by qPCR using the ABI 7000 Sequence Detection System with 1 cycle 10-min at 95°C and 40 cycles 15 sec at 95°C and 1 min at 60°C with Qiagen/SA Biosciences SYBR Green Master Mix as described previously [13], using 20 ng DNA and the following specific forward and reverse primers: *P*. *distasonis* (TGATCCCTTGTGCTGCT and ATCCCCCTCATTCGGA) [79], *P*. *falsenii* (CGTGGACCAAAGTTATTTCGGTAGA and AAACAACCCCTCATTTCTCA) [80], *A*. *finegoldii* (GTACTAATTCCCCATAACATTCGAG and CTAATACAACGCATGCCCATCTT) [79], *B*. *eggerthii* (GTCATATTAACGGTGGCG and GGGTTBCCCCATTCGG) [79], and universal primers for all *Eubacteria* (ACTCCTACGGGAGGCAGCAGT and ATTACCGCGGCTGCTGGC) [13]. The amounts of DNA for each bacterial species for each mouse were calculated using comparative cycle threshold method with *Eubacteria* as a control.

### Statistical analyses

The significance of differences between the numbers of recovered bacterial species and OTUs and between the numbers of taxonomic units with increased and decreased abundance was determined by binomial test if npq < 9 or by z-test if npq ≥ 9 using Microsoft Excel. The significance of differences between all other quantitative results presented as means ± SEM were determined by the Student’s *t*-test using Microsoft Excel. The significance of differences between mouse survivals were analyzed using Chi-square test. The *N* and *P* values are indicated in the figures and tables; *P* ≤ 0.05 was considered significant. The heatmaps were generated using Java TreeView and represent mean fold changes in bacterial abundance relative to WT, after converting <1 ratios to negative fold difference using the formula: (-1)/ratio.

## Supporting Information

S1 Fig α-diversity in stool microflora in WT and *Pglyrp*-deficient mice. (**A**) Total numbers of species, numbers of species/mouse, Shannon diversity index, and Shannon equitability index for bacterial species. (**B**) Total numbers of OTUs and numbers of OTUs/mouse. The results are means ± SEM or totals; *N* = 12 mice/strain; *, *P*<0.05; **, *P*<0.001 *Pglyrp*-deficient *versus* WT.
(TIF)

S1 Table Bacterial classes in stools with significantly different abundance in *Pglyrp*-deficient mice than in WT mice. (XLS)

S2 Table Bacterial orders in stools with significantly different abundance in *Pglyrp*-deficient mice than in WT mice. (XLS)

S3 Table Bacterial families in stools with significantly different abundance in *Pglyrp*-deficient mice than in WT mice. (XLS)

S4 Table Bacterial genera in stools with significantly different abundance in *Pglyrp*-deficient mice than in WT mice. (XLS)

S5 Table Bacterial species in stools with significantly different abundance in *Pglyrp*-deficient mice than in WT mice. (XLS)

S6 Table Bacterial OTUs in stools with significantly different abundance in *Pglyrp*-deficient mice than in WT mice. (XLS)
