# Supplementary figures and images for: Pglyrp-Regulated Gut Microflora Prevotella falsenii, Parabacteroides distasonis and Bacteroides eggerthii Enhance and Alistipes finegoldii Attenuates Colitis in Mice
Source: PLoS One. 2016 Jan 4;11(1):e0146162. doi: 10.1371/journal.pone.0146162 (PMC4699708; doi:10.1371/journal.pone.0146162)

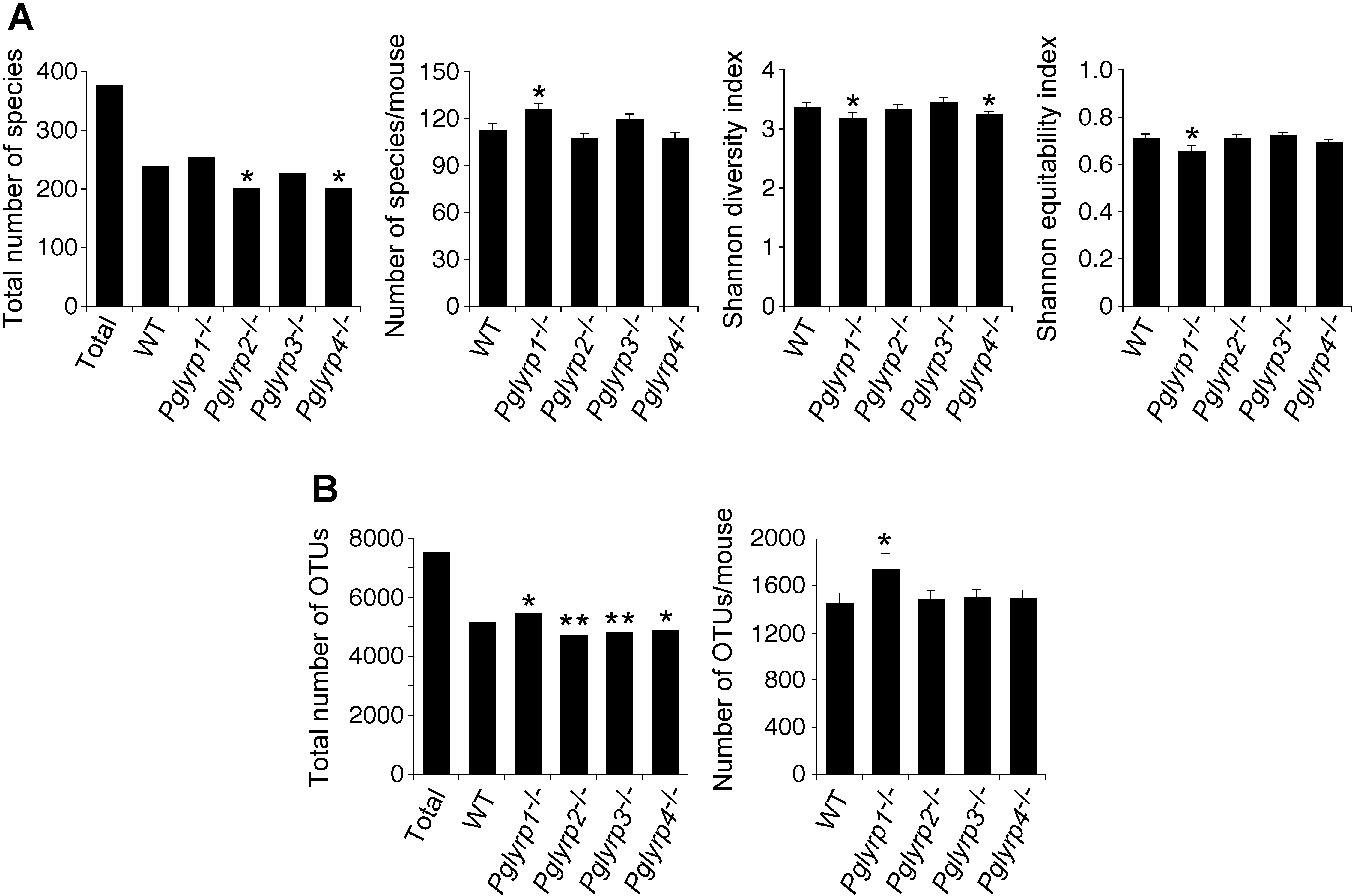

Supplement: S1 Fig — (A) Total numbers of species, numbers of species/mouse, Shannon diversity index, and Shannon equitability index for bacterial species. (B) Total numbers of OTUs and numbers of OTUs/mouse. The results are means ± SEM or totals; N = 12 mice/strain; *, P<0.05; **, P<0.001 Pglyrp-deficient versus WT. (TIF) [file pone.0146162.s001.tif]
